# Characterizing the Bacterial Microbiome of the Invasive Vector *Aedes albopictus* in Hungary: A Pilot Study Using Oxford Nanopore Sequencing

**DOI:** 10.1155/ijm/1956331

**Published:** 2026-02-18

**Authors:** Kornélia Kurucz, Camille Philippe, Ágota Ábrahám, Myriam Kratou, Elianne Piloto-Sardiñas, Dasiel Obregon, Lianet Abuin-Denis, Andrea Kovács-Valasek, Alejandro Cabezas-Cruz

**Affiliations:** ^1^ National Laboratory of Virology, Szentágothai Research Centre, University of Pécs, Pécs, Hungary, pte.hu; ^2^ Institute of Biology, Faculty of Sciences, University of Pécs, Pécs, Hungary, pte.hu; ^3^ Sciensano, Belgian Institute for Health, Brussels, Belgium, sciensano.be; ^4^ Laboratory of Immunology, Department of Translational Physiology, Infectiology and Public Health, Faculty of Veterinary Medicine, Ghent University, Merelbeke, Belgium, ugent.be; ^5^ Laboratory of Microbiology, National School of Veterinary Medicine of Sidi Thabet, University of Manouba, Manouba, Tunisia, uma.rnu.tn; ^6^ Direction of Animal Health, National Center for Animal and Plant Health, Carretera de Tapaste y Autopista Nacional, San José de las Lajas, Cuba; ^7^ Anses, INRAE, Ecole Nationale Vétérinaire d′Alfort, UMR BIPAR, Laboratoire de Santé Animale, Maisons-Alfort, France, anses.fr; ^8^ School of Environmental Sciences, University of Guelph, Guelph, Ontario, Canada, uoguelph.ca; ^9^ Animal Biotechnology Department, Center for Genetic Engineering and Biotechnology, Havana, Cuba, cigb.edu.cu

## Abstract

*Aedes albopictus* has recently established self‐sustaining populations in Hungary, but its microbiota—which may influence vector competence—remains poorly understood. We used Oxford Nanopore long‐read sequencing for full‐length 16S rRNA gene profiling of adult *Ae. albopictus* from two urban sites, Pécs and Barcs. Each location contributed 10 specimens, with contamination controls rigorously applied. Diversity metrics and co‐occurrence network analyses were performed using QIIME2, SparCC, and NetCoMi, with robustness assessed via simulated node removal and addition. Sequencing depth was sufficient to saturate rarefaction curves. Although alpha and beta diversity did not differ significantly between sites, the Pécs population exhibited greater taxonomic richness (100 unique taxa vs. 61 in Barcs) and denser, more clustered networks. Only 15 genera were shared, with *Wolbachia* dominating both communities. Networks differed in central taxa and structural properties: Pécs retained higher connectivity and shorter paths under perturbation, suggesting greater resilience. Removal of conserved taxa revealed location‐specific impacts on network stability, with Pécs more vulnerable to the loss of key genera. Negative interactions and compensatory taxa emerged post‐removal, indicating distinct reconfiguration strategies. Our findings highlight marked local variation in microbiome structure and robustness, even across a 65‐km gradient. These results establish a high‐resolution baseline for assessing how microbiota shape *Ae. albopictus* vector potential, informing microbiome‐based control strategies tailored to regional contexts.

## 1. Introduction

Mosquitoes, particularly those of the genera *Aedes*, *Culex*, and *Anopheles*, are major vectors of infectious diseases, transmitting arboviruses such as dengue (DENV), chikungunya (CHIKV), Zika (ZIKV), and West Nile virus (WNV), as well as malaria parasites and filarial nematodes [1, 2]. In Europe, the emergence and dispersal of new vector species represent a major public health concern. Although various exotic mosquito species are regularly introduced, three are currently considered established: the Asian tiger mosquito (*Aedes albopictus*), the Japanese bush mosquito (*Aedes japonicus*), and the Korean bush mosquito (*Aedes koreicus*) [3, 4]. The invasive success of *Ae*. *albopictus* is largely attributed to ecological traits such as drought‐resistant eggs and a high adaptive capacity, which enhance survival, facilitate rapid dispersal, and support establishment in new environments. Although hibernation is not exclusive to invasive species, since native *Aedes* species also exhibit this behavior, the true driver behind its expansion is the mosquito′s ability to adjust to novel environmental conditions. This adaptability enables *Ae. albopictus* to colonize diverse habitats and persist despite climatic fluctuations [5]. The spread of these species occurs across multiple spatial scales and is closely linked to human mobility. At the continental level, eggs are passively transported via goods like used tires and ornamental plants (e.g., lucky bamboo) on cargo ships, whereas at national and regional levels, adult mosquitoes are dispersed primarily through ground transportation, especially along highways [6].

Among these species, *Ae. albopictus*, native to Asia, stands out as one of the most invasive mosquitoes worldwide. In Europe, it was first detected in Italy in 1990 and has since spread extensively across Southern and Central Europe [3, 7, 8]. It was reported in the north of the Alps in Germany in 2007 [7], and more recently, the northernmost established population in Europe was observed in Berlin [9]. Moreover, *Ae. albopictus* is known for its ecological plasticity, aggressive daytime biting behavior, and ability to feed on both humans and animals, making it an effective bridge vector for zoonotic pathogens [10]. Its establishment in Europe has enabled the local transmission of arboviruses previously considered exotic. For example, a CHIKV outbreak in Italy in 2007, triggered by a viremic traveler from India, resulted in over 200 clinical cases [11]. Subsequent autochthonous transmissions were recorded in France in 2010, 2014, and 2017, with another major outbreak in Italy in 2017 [12–14]. In addition, climate warming is expected to significantly alter the risk of transmission for vector‐borne pathogens in Europe [15]. Rising temperatures will further facilitate the spread of the thermophilic *Ae. albopictus* [16]. In recent years, a marked increase in the detection of mosquito populations has been observed at multiple sites north of the Alps [9]. The greatest future risk is anticipated for CHIKV outbreaks. As CHIKV can be transmitted even at relatively low temperatures, its spatial risk in Europe is currently more constrained by the distribution and density of *Ae. albopictus* populations than by climate alone [17].

All of this has had a substantial impact on mosquito control strategies as well. Historically, mosquito surveillance and control efforts in Europe were limited to areas with severe mosquito nuisance. However, this shifted to the establishment of invasive mosquito species and the emergence of vector‐borne pathogens. Although a wide array of control tools is available, and their implementation within integrated vector management is established in many countries, mosquito control still faces significant and evolving challenges. Because of the limited long‐term success of conventional strategies, there is a growing shift in focus toward reducing vector competence of mosquitoes rather than solely suppressing their population size. In this context, the recognition that bacterial symbionts of mosquitoes may significantly influence their capacity to transmit given pathogens opened new perspectives in vector control [18, 19].

The physiology of *Aedes* mosquitoes is profoundly influenced by their microbiota, which affects critical biological processes such as reproduction, egg production, blood digestion [20], and overall survival [21, 22]. Certain bacterial taxa enhance mosquito immunity by producing antimicrobial peptides, whereas others aid in blood meal digestion. The composition of the microbiota can also modulate host–pathogen interactions and immune regulation [23] by either promoting or inhibiting pathogen development within the mosquito, thereby impacting its vectorial capacity and potential for pathogen transmission [24, 25]. In light of this, manipulating the mosquito microbiome could be a promising strategy against vector‐borne diseases. The most well‐documented case is *Wolbachia*, an intracellular bacterium capable of reducing DENV, ZIKV, and WNV replication within mosquitoes [26–28]. Beyond *Wolbachia*, other bacterial genera such as *Asaia*, *Serratia*, and *Enterobacter* have also been implicated in modulating mosquito immunity and interfering with pathogen development [29, 30]. As a potential biocontrol tool, *Wolbachia* has received considerable attention due to its ability to induce cytoplasmic incompatibility, a reproductive manipulation first described by Yen and Barr [31] in *Culex* mosquitoes, where incompatible sperm–egg pairings fail to produce viable offspring. Furthermore, the introduction of exogenous *Wolbachia* strains into *Ae. albopictus* populations has shown promise in disrupting pathogen transmission and reducing vector competence [26]. However, most natural populations of *Ae. albopictus* already host endogenous *Wolbachia* strains [32], and interactions between coexisting strains can vary significantly, potentially affecting the outcomes of microbiome‐based control strategies [26].

Since the bacterial microbiome of mosquitoes is of critical importance for vector biology and disease transmission dynamics, understanding the composition and structure of mosquito‐associated microbiota is a crucial step toward developing novel vector control strategies. In particular, the structure of these microbial communities varies significantly depending on the mosquito species, developmental stage, sex, and environmental factors [33]. Traditional methods such as culture‐based techniques or short‐read sequencing platforms have been widely used in microbial community analysis; however, these methods are often limited by scalability (incomplete taxonomic resolution, amplification bias) and time‐consuming workflows. However, real‐time and long‐read sequencing using Oxford Nanopore Technologies (ONT) has transformed the landscape of microbiome and pathogen surveillance [34]. ONT platforms enable simultaneous species identification, microbiota profiling, and detection of endemic, epidemic, or novel viruses in mosquito populations through integrated amplicon‐based DNA barcoding and metagenomic sequencing [34]. Building on this enhanced microbiome characterization, emerging microbiota‐based control strategies, such as antimicrobiota vaccines, have shown promise in modulating vector competence. Recent studies have demonstrated that targeting specific bacterial taxa can shift the microbial composition of *Ae. albopictus*, with impacts on both pathogen transmission and mosquito fitness [35]. For instance, Mateos‐Hernández et al. [35] found that antimicrobiota vaccination increased fecundity and egg‐hatching rates, whereas Aželytė et al. [36] reported a reduced avian malaria infection following similar interventions. These findings suggest that immunologically driven alterations of the mosquito microbiome may serve as a viable and complementary strategy for controlling vector‐borne diseases.

Building on recent advances in real‐time sequencing and microbiota‐based vector control strategies, this study focuses on the bacterial microbiome characterization of *Ae. albopictus* using ONT, in order to gain a more comprehensive, high‐resolution profile of the mosquito microbiota. Very limited data exist on the microbial ecology of these local populations of *Ae. albopictus*. Since the microbiome can influence mosquito immunity, pathogen susceptibility, and even vector competence, understanding the bacterial communities of *Ae. albopictus* in Hungary could offer key insights into their local adaptation, ecological fitness, and vectorial potential [10]. Given that microbiota structure is influenced by regional environmental conditions, understanding the bacterial communities associated with *Ae. albopictus* is critical for assessing potential variations in vector competence. Here, we used ONT′s portable, high‐throughput sequencing platform to characterize the bacterial microbiome of *Ae. albopictus* from two populations in southwestern Hungary: in the city of Pécs and Barcs. Our study is aimed at (i) assessing microbial diversity, (ii) identifying dominant bacterial genera with potential implications for pathogen transmission, and (iii) analyzing microbial co‐occurrence networks to evaluate community structure and stability. By integrating microbiome profiling with ecological network and robustness analyses, this study provides a foundational understanding of mosquito‐associated microbiota in Hungary and its possible role in shaping vector competence.

## 2. Materials and Methods

### 2.1. Collection of *Ae*. *albopictus* Mosquito Samples

Mosquito specimens were collected from urban habitats in the cities of Pécs (Baranya County) and Barcs (Somogy County), about 65 km apart, representing two independent *Ae. albopictus* populations from the southwestern region of Hungary. Sampling was conducted in the frame of local monitoring programs, run in residential areas between May and October in 2022 and 2023, using BG‐Sentinel traps (Biogents, Germany) baited with BG lure and CO_2_ as attractants and operated once per week overnight. The collected mosquitoes were morphologically identified at the species level using standard taxonomic keys, then stored native (without any medium) at −20°C until further processing. Considering the pilot nature of our investigation, from both populations, 10 adult *Ae. albopictus* individuals were randomly selected for further microbiome analysis.

### 2.2. Nucleic Acid Extraction From Mosquito Samples

Each mosquito was individually homogenized in 200 *μ*L of sterile water using sterile quartz sand and tissue disruptor sticks. The homogenate was centrifuged to pellet debris, and DNA was extracted from the supernatant using the Quick‐DNA Miniprep Plus Kit (Zymo Research, Irvine, United States), following the manufacturer′s protocol. In addition, in order to ensure high‐quality DNA recovery, all extractions were performed under sterile conditions, and appropriate precautions were taken to minimize contamination. To confirm that no contamination was introduced during the extraction process, four controls consisting of sterile water only were included and processed in the same way as the samples.

### 2.3. Library Preparation and Nanopore Sequencing

The sequencing library was prepared using the 16S Barcoding Kit (SQK‐16S114.24, ONT, United Kingdom) according to the manufacturer′s instructions. DNA concentration was quantified using the Qubit 1X dsDNA HS Assay Kit (Thermo Fisher Scientific, United States) on a Qubit 4 Fluorometer, ensuring that each sample contained 10 ng of input DNA in a 15‐*μ*L volume for barcoding PCR. In the barcoding PCR step, the 16S rRNA gene was amplified using barcoded primers provided in the ONT sequencing kit. Cycling parameters were set as follows: initial denaturation at 95°C for 1 min, followed by 35 cycles of denaturation at 95°C for 20 s, annealing at 55°C for 30 s, and extension at 65°C for 2 min. A final extension at 65°C for 5 min ensured a complete amplicon synthesis. After amplification, reactions were held at 4°C to preserve sample integrity prior to downstream processing. To amplify low‐biomass DNA from individual mosquitoes, we increased the number of PCR cycles from the standard 25 to 35, as suggested by the library preparation kit′s protocol to improve amplification yield.

Post‐PCR DNA concentrations were remeasured using the same Qubit method to ensure quality control. The resulting amplicons were pooled in equimolar ratios, purified using AMPure XP Beads (Beckman Coulter, United States) at a 1:1 ratio, and eluted into 30 *μ*L of Elution Buffer (SQK‐16S114.24, ONT, United Kingdom). A single flow cell (R10.4.1) was used for all samples, following the recommendations of the sequencing kit and leveraging its capacity to accommodate up to 24 barcoded samples and controls.

For final library construction, 1 *μ*L of a diluted adapter mix was added to 50 fmol of pooled samples, as per the ONT protocol. A single flow cell (R10.4.1) was used for all samples, following the recommendations of the sequencing kit and leveraging its capacity to accommodate up to 24 barcoded samples and controls. The sequencing library was then loaded onto an R10.4.1 flow cell (FLO‐MIN114, ONT, United Kingdom) and sequenced using a MinION Mk1B (ONT, United Kingdom) device for 72 h to ensure sufficient depth, based on prior runs with shorter durations that failed to achieve adequate coverage.

To account for potential contamination, four negative controls containing sterile water were processed alongside the mosquito samples through DNA extraction, library preparation, and sequencing. These controls were handled identically to biological samples and were analyzed to confirm that detected microbial signatures originated from the mosquito specimens rather than external sources.

### 2.4. Sequencing Data Processing and Taxonomic Classification

All sequencing data processing was conducted on a Ubuntu 22.04 LTS operating system. Raw sequencing data were initially stored in FAST5 format during the sequencing run. After completion, basecalling was performed using Guppy Basecaller (v6.5.7, ONT), applying the super‐accurate basecalling model with the configuration file.

Following basecalling, demultiplexing was conducted with Guppy Barcoder (v6.5.7, ONT) using barcode sequences from the 16S Barcoding Kit, ensuring accurate assignment of reads to their respective samples.

To assess data quality, NanoPlot (v1.41) was used for initial quality control. Based on these results, reads were filtered with NanoFilt (v2.8), retaining only sequences between 1000 and 2000 base pairs (bp) to ensure high‐quality 16S rRNA gene reads.

The reads were classified to create the taxa table using the EPI2ME 16S Workflow in EPI2ME labs software (https://github.com/epi2me-labs/wf-16s), with Minimap2 for alignment and the SILVA database (SILVA_138_1) [37] for taxonomic classification. Rarefaction curves (Figure S1) were generated in RStudio [38], these curves permit assessing sequencing depth. Contaminant sequences were identified using the Decontam package [39] in RStudio [38], applying the prevalence‐based method, which detects contaminants by comparing their occurrence in true samples versus negative controls. A probability threshold of 0.5 was used to classify taxa as contaminants.

### 2.5. Sequencing Data Availability

The data that support the findings of this study are openly available in Sequence Read Archive (SRA) at https://www.ncbi.nlm.nih.gov/sra, Reference Number PRJNA1290917.

### 2.6. Microbial Diversity and Composition Analysis

To evaluate differences in bacterial diversity within samples, alpha and beta diversity metrics were calculated using the q2‐diversity plugin in QIIME2 [40]. Alpha diversity was assessed using observed features (taxa richness), Pielou′s evenness (taxa distribution), and Shannon entropy (combined richness and evenness). Statistical significance was determined using the Kruskal–Wallis test (*p* ≤ 0.05), and results were visualized with GraphPad Prism v9.4.1 (681) (GraphPad Software Inc.). Bray–Curtis dissimilarity index was employed to evaluate beta diversity differences, with calculation based on a PERMANOVA test (*p* < 0.05) and Past 4 Version 4.06b. Beta dispersion was calculated using the betadisper function and the Vegan package [41] implemented in RStudio [38] (RStudio), using an ANOVA test (*p* ≤ 0.05) as statistical analyses. Cluster analysis was performed with the Jaccard coefficient of similarity using Vegan [41] implemented in RStudio (RStudio). Next, unique and shared taxa across study areas were visualized using a Venn diagram generated with the Venn webtool (https://bioinformatics.psb.ugent.be/webtools/Venn/).

### 2.7. Co‐Occurrence Network Analysis

To further investigate the microbial community structure and interactions, co‐occurrence network analysis was employed to compare the architecture and node hierarchy between networks of *Ae. albopictus* across both localities. Microbial co‐occurrence networks were inferred from genus‐level taxonomic profiles to visualize community structure and interactions. In the resulting networks, nodes represent bacterial genera, and edges indicate significant correlations between them. Node color reflects modularity class, whereas node size corresponds to eigenvector centrality, highlighting the relative influence of each taxon. Significant associations either positive (≥ 0.5) or negative (≤ −0.5) were identified using the Sparse Correlations for Compositional data (SparCC) method [42], implemented in RStudio [38]. Visualization and measurement of topological features [i.e., number of nodes and edges, network diameter, modularity, average degree, weighted degree, clustering coefficient, and number of modules] of the networks were performed using Gephi v0.10 [43].

### 2.8. Local Connectivity of Conserved Shared Taxa

Using Gephi v0.10 [43], subnetworks were constructed to visualize the local connectivity of conserved shared bacterial genera within the global microbial networks of the Pécs and Barcs. This approach allowed for the comparative analysis of taxonomic interaction patterns and assemblage variation between both communities, focusing on differences in the specificity of the taxa in direct association and the number of interactions.

### 2.9. Network Comparison

Network comparisons were performed using the “NetCoMi” package [44] in R v4.3.1 (R Core Team, 2023), using RStudio [38]. NetCoMi facilitates the alignment of microbial association networks by matching nodes (taxa) and edges (co‐occurrence relationships) based on their topological features, enabling the detection of shared structural patterns. Besides, with the interest to quantify network dissimilarities, the Jaccard index was calculated for key centrality measures, including degree, betweenness, closeness, and eigenvector centrality. This index assesses overlap in the sets of highly central nodes defined as those above the empirical 75th percentile and ranges from 0 (completely different sets) to 1 (sets are equal). Two‐tailed *p* values, *P* (*J* ≤ *j*) and *P* (*J* ≥ *j*), were used to determine whether the observed Jaccard indices significantly deviated from random expectations [45].

### 2.10. Robustness Analysis Based on Node Removal and Addition

To assess the robustness of microbial co‐occurrence networks, the effects of targeted node removal on network connectivity were examined using the “NetSwan” package (Network Strengths and Weaknesses Analysis) [46] in R v.4.3.1 (R Core Team, 2023), using RStudio [38]. Direct attacks involved the sequential removal of a proportion of nodes based on specific criteria: betweenness centrality, degree centrality, and cascading. In the betweenness‐based approach, nodes with the highest betweenness centrality were progressively removed. Similarly, in the degree‐based approach, removal was prioritized for nodes with the highest degree centrality. However, the cascading method involved the iterative removal of high‐betweenness nodes with recalculation of centrality values after each step.

In addition, a node addition analysis was performed in RStudio [38] based on the approach described by Freitas et al. [47]. Random nodes were incrementally introduced into the existing network, and structural changes were assessed by measuring the Largest Connected Component (LCC) and Average Path Length (APL). To ensure a robust evaluation, multiple simulations were conducted with different node sets (100, 300, 500, 700, and 1000 nodes). Results were visualized using GraphPad Prism v9.4.1 (GraphPad Software, San Diego, California, United States). Statistical significance for LCC and APL was tested using the Wilcoxon signed‐rank test, with *p* values adjusted via the Benjamini–Hochberg method to control the false discovery rate. Bootstrapping was applied to estimate confidence intervals, and significance was defined as *p* < 0.05.

### 2.11. Network Modeling and Robustness Testing in Response to Shared Taxa Removal

To analyze topological variations in microbial networks and assess community stability following the removal of shared taxa between the two conditions (Pécs and Barcs), a simulation was conducted. In this simulation, all read counts corresponding to the shared taxa, *Pantoea* (Pécs woP/Barcs woP), *Stenotrophomonas* (Pécs woS/Barcs woS), *Delftia* (Pécs woD/Barcs woD), and *Alcaligenes* (Pécs woA/Barcs woA), were set to zero across all samples from both groups. Microbial co‐occurrence networks were reconstructed using the SparCC method [42], implemented in RStudio [38]. Topological metrics were computed and compared between the original and taxa‐depleted networks (e.g., Pécs vs. Pécs woP and Barcs vs. Barcs woP) [the number of nodes and edges, network diameter, modularity, average degree, weighted degree, and clustering coefficient]. To further assess community structure and stability, variations in the nestedness patterns of condition‐specific taxa were analyzed.

Network robustness was evaluated by simulating both directed attacks (based on betweenness centrality, degree centrality, and cascading failure) and random attacks. The resulting loss of connectivity and structural integrity was measured to assess the resilience of microbial communities to targeted and stochastic disruptions. These simulations were performed using the NetSwan package [46] in R Version 4.3.1 (R Core Team, 2023), implemented in RStudio [38].

## 3. Results

In general, sequencing was successfully performed on 10 mosquitoes from Pécs and 10 from Barcs, incorporating replicated negative controls. Sequencing depth was sufficient to capture the microbial diversity, as indicated by the rarefaction curves (Figure S1). A taxa table showing the read counts per sample is provided in Table S1.

### 3.1. Microbial Diversity and Composition in the Mosquito Microbiome From Pécs and Barcs

Alpha diversity analysis, based on various metrics, showed a slightly higher number of observed features in Pécs compared with Barcs, although the difference was not statistically significant (Kruskal–Wallis, *p* = 0.29; Figure 1a). In addition, Pielou′s evenness (*p* = 0.36) and Shannon entropy (*p* = 0.40) were also higher in Pécs, but similarly, the differences were not significant (Figure 1b,c). Similarly, beta diversity analysis based on the Bray–Curtis dissimilarity index revealed compositional differences in microbial communities between Pécs and Barcs, as visualized by Principal Coordinates Analysis (PCoA; Figure 1d). However, these differences were not statistically significant according to PERMANOVA (*p* = 0.06). Moreover, hierarchical clustering based on the Jaccard distance (Figure 1e) revealed partial separation between samples from Pécs and Barcs, with some clustering by location but also overlapping patterns between the two groups (Figure 1e).

Figure 1Comparison of microbial diversity and community composition of mosquito microbiome from Pécs and Barcs. Comparison of alpha diversity between Pécs and Barcs groups: (a) observed features (Kruskal–Wallis test, *p* = 0.29), (b) Pielou′s evenness (Kruskal–Wallis test, *p* = 0.36), and (c) Shannon entropy (Kruskal–Wallis test, *p* = 0.40). (d ) Comparison of beta diversity with Bray–Curtis dissimilarity index between Pécs and Barcs groups. Beta dispersion of two sets of samples (pairwise comparison). Small circles and triangles represent samples, and ellipses represent centroid position for each group. PERMANOVA test was performed and showed that beta dispersion of the two sets of samples is not significantly different (*p* = 0.06). (e) Hierarchical clustering of samples based on microbial composition using Jaccard distance. Samples from Pécs (blue) and Barcs (red) show distinct clustering patterns. (f) Venn diagram displaying the number of shared and unique taxa between Pécs and Barcs. The overlap represents the taxa found in both locations.

**Figure:**
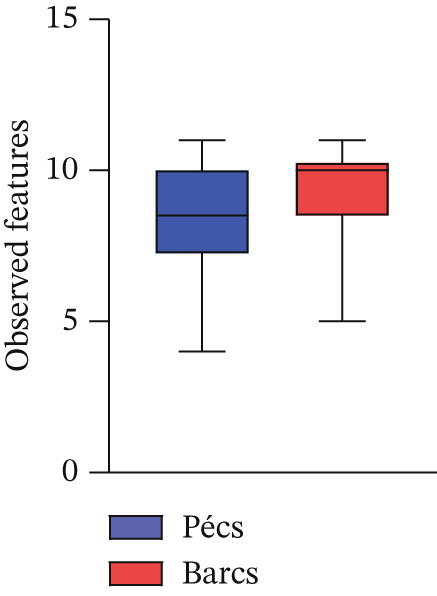
(a)

**Figure:**
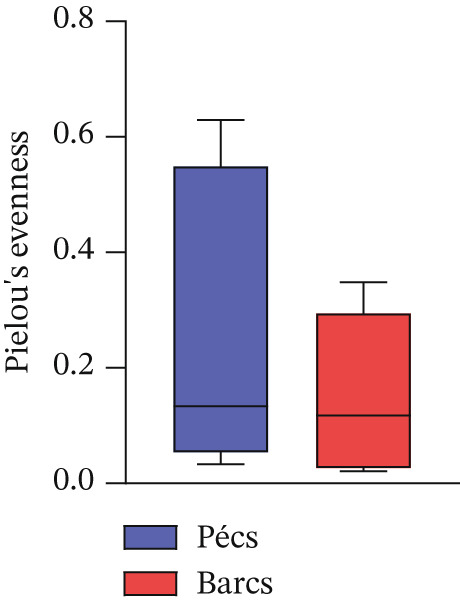
(b)

**Figure:**
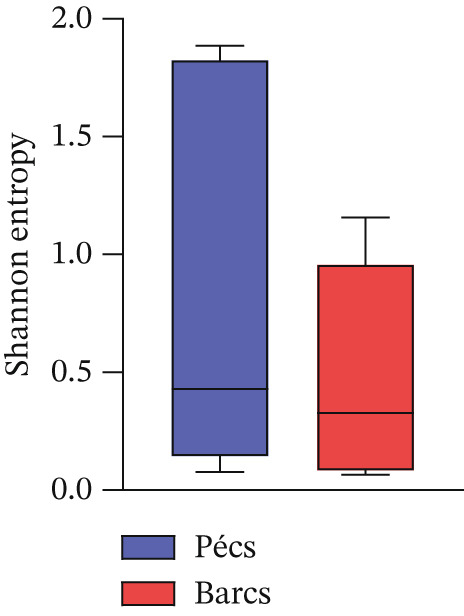
(c)

**Figure:**
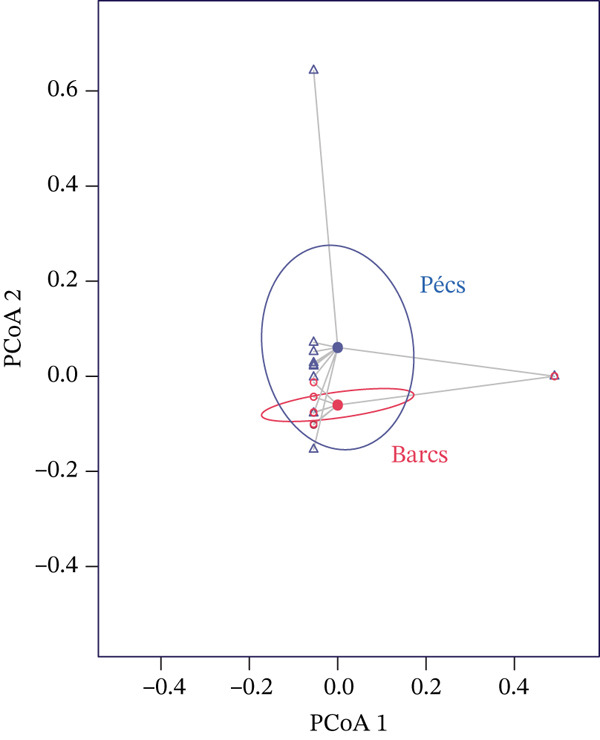
(d)

**Figure:**
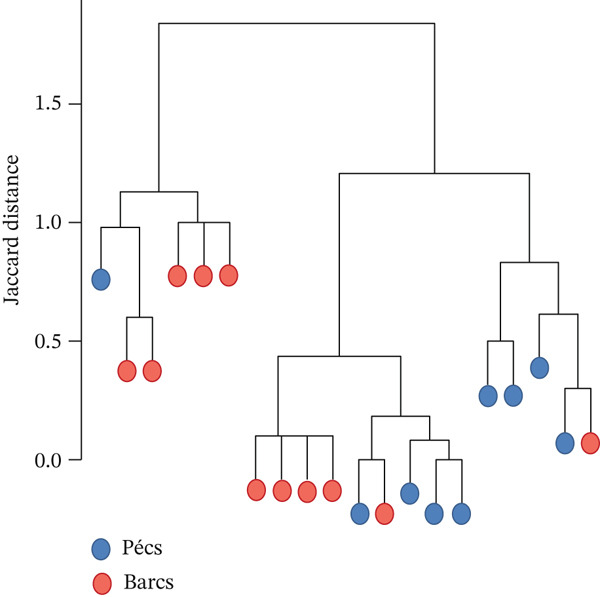
(e)

**Figure:**
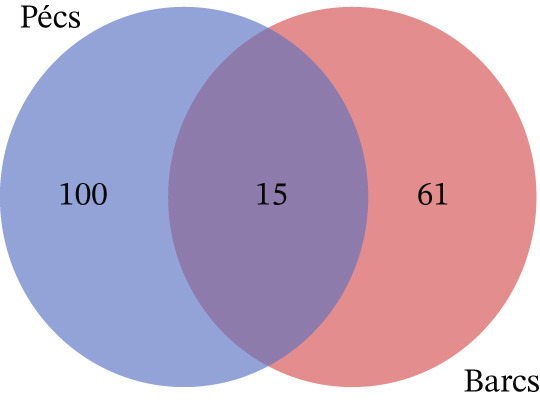
(f)

Although not statistically significant, the trend toward greater richness, evenness, and Shannon entropy observed in the microbial community associated with *Ae. albopictus* in Pécs suggests a more evenly distributed biodiversity. These results suggest a less hierarchical microbiota, which could lead to a community with greater ecological plasticity and, consequently, greater resilience to external disturbances. In contrast, the limited beta diversity between the Pécs and Barcs samples suggests a high degree of compositional homogeneity. Taken together, these findings imply that while individual mosquitoes in Pécs harbor more diverse and evenly distributed microbiotas, the overall community composition remains relatively constant across both locations.

Furthermore, compositional analysis identified 15 taxa shared between the two populations, with 100 taxa unique to mosquitoes from Pécs and 61 unique to mosquitoes from Barcs (Figure 1f, Table S2). These findings reinforce the existence of a shared core microbiota but also highlight location‐specific microbial characteristics that could be influenced by environmental or host‐related factors. Interestingly, among the shared taxa between the *Ae. albopictus* populations from Pécs and Barcs, some bacterial genera were consistently detected, including *Wolbachia*, *Acinetobacter*, *Pantoea*, *Stenotrophomonas*, *Delftia*, *Leuconostoc*, and *Alcaligenes*. The presence of these specific genera in geographically distinct locations is particularly noteworthy, as several of them have been previously reported in association with mosquitoes and may be involved in host–microbe interactions. It suggests a set of stable taxa that could represent part of the conserved core microbiota of *Ae. albopictus*.

### 3.2. Microbial Community Assembly Comparison Among the Mosquito Microbiome From Pécs and Barcs

The co‐occurrence network analysis revealed distinct structural differences between the locations. The network from Pécs exhibited a highly interconnected structure with a greater number of nodes and edges, characterized by a central cluster of strongly co‐occurring taxa, whereas the network from Barcs was more fragmented, consisting of smaller and more distinct clusters of microbial taxa (Figure 2a). In addition, Pécs showed elevated average degree and clustering coefficient values (Table 1), whereas Barcs displayed a higher modularity score, reflecting the presence of more distinct microbial subgroups with lower connectivity between them (Table 1). This suggests that microbial communities in Pécs may be more cohesive and interactive compared to those in Barcs.

Figure 2Microbial community assembly in the mosquito microbiome from Pécs and Barcs. (a) Network analysis of microbial co‐occurrence patterns in mosquito samples from Pécs and Barcs. Nodes represent the taxa, and the colors are based on modularity class metric where equal color means modules of co‐occurring taxa. The size of the nodes is proportional to the eigenvector centrality of each taxon. Only positive associations are shown in blue (SparCC > 0.5), whereas negative correlations were excluded from the network for clarity. (b) Differential network comparison of Pécs and Barcs groups. Only positive associations are shown. Edge color (green) indicates the presence and strength of these positive interactions.

**Figure:**
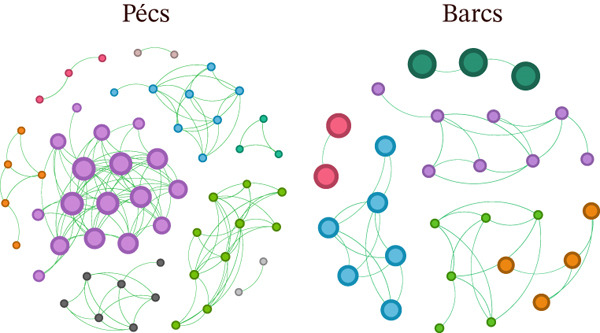
(a)

**Figure:**
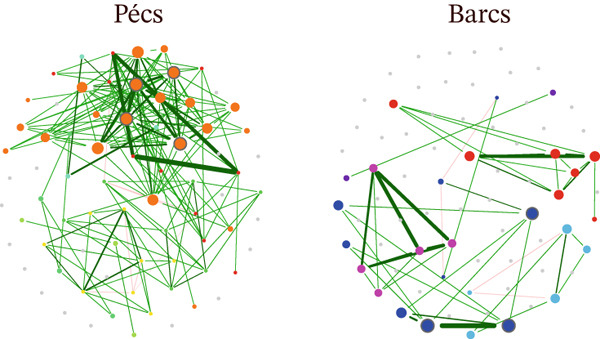
(b)

**Table 1:** Topological features of the taxonomic networks of each ecoregion.

Network features	Pécs	Barcs
Number of nodes	57	29
Number of edges	146	41
Positive interactions	100%	100%
Negative interactions	0%	0%
Modularity	0.691	0.749
Network diameter	3	3
Average degree	5.123	2.828
Weighted degree	3.156	1.841
Clustering coefficient	0.82	0.771
Number of modules	8	5

Interestingly, despite the high abundance of *Wolbachia* and *Acinetobacter* in both microbial communities, *Wolbachia* was absent from both the Pécs and Barcs networks, whereas *Acinetobacter* was absent only from the Pécs network (Figure 2a). This exclusion may be due to competitive selective pressures exerted by other ecologically dominant taxa or reflect a functional dissociation whereby highly abundant bacteria do not require frequent interactions to maintain their presence and stability within the microbial community.

NetCoMi was used to assess dissimilarities in local network centrality measures between the two networks (Figure 2b). The Jaccard index was calculated for degree, betweenness centrality, closeness centrality, and eigenvector centrality (*J* *a* *c* *c* = 0, lowest similarity; *J* *a* *c* *c* = 1, highest similarity). The results revealed structural differences between the networks of Pécs and Barcs. The Pécs network was denser, indicating a more interconnected microbiome, whereas the Barcs network appeared more fragmented (Figure 2b). In addition, the Jaccard index showed weak similarity between the microbiomes of the two areas (Table 2). The *P* (≤ Jacc) value confirmed that the microbiome differences for degree, closeness centrality, eigenvector centrality, and hub taxa were nonrandom and statistically significant, whereas the difference in betweenness centrality was not significant (Table 2).

**Table 2:** Jaccard index results for Pécs and Barcs networks.

Centrality measures	Jaccard index	*P* (≤ Jacc)	*P* (≥ Jacc)
Degree centrality	0.106	0∗∗∗	1
Betweenness centrality	0.150	0.06	0.98
Closeness centrality	0.106	0∗∗∗	1
Eigenvector centrality	0.123	0∗∗∗	1
Hub taxa	0.120	0∗∗∗	1

**Figure 3:**
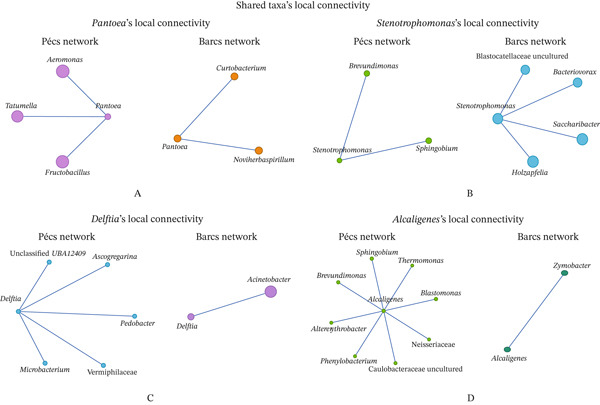
Comparison of the assembly patterns of shared taxa between Pécs and Barcs groups. Local connectivity of (A) *Pantoea*, (B) *Stenotrophomonas*, (C) *Delftia*, and (D) *Alcaligenes.* Node colors are based on modularity class metric, whereas the size of the nodes is proportional to the eigenvector centrality value of each taxon. The colors of the edges represent strong positive (blue) or negative (red) correlations (SparCC > 0.5 or < −0.5).

The topological features, graphical representations of microbial networks, and network comparisons revealed marked differences in microbial community assembly patterns between the two mosquito populations. However, this variability could extend to shared and conserved bacterial taxa within the *Ae. albopictus* microbiota. To explore this possibility, four shared genera—*Pantoea* (Figure 3A), *Stenotrophomonas* (Figure 3B), *Delftia* (Figure 3C), and *Alcaligenes* (Figure 3D)—were selected and analyzed by comparatively assessing their local connectivity between both populations. This approach allowed for a targeted examination of differential integration and ecological functions within distinct network contexts. A consistent pattern was observed, as all bacterial genera analyzed, except *Stenotrophomonas* (Figure 3B), showed a reduced number of interactions within the microbial community associated with the Barcs group compared with Pécs. This reduction aligns with a broader loss of biodiversity, lower complexity of interactions, and a general deterioration of network architecture. The contrasting topological features suggest a more fragmented and less integrated microbial community in Barcs, which could reflect ecological instability or reduced functional redundancy. On the other hand, the increase in interactions in *Stenotrophomonas* (Figure 3B) under depletion conditions suggests compensatory behavior or a functional reorganization of the network, where this genus assumes a more central role in the face of biodiversity and interaction loss.

### 3.3. Stability of Mosquito‐Associated Microbial Communities From Pécs and Barcs

Robustness to node removal (Figure 4a) shows that the two networks are similarly affected by node removal. However, a more specific analysis revealed that a larger number of node removals were required to achieve 80% disconnection in the Barcs network in cascading and degree‐based attack scenarios, whereas fewer removals were required in betweenness attacks (Figure 4a). These patterns indicate a structurally fragile network with limited redundancy in Barcs.

Figure 4Robustness comparison against removal and addition of nodes between Pécs and Barcs groups. (a) Comparison between Pécs and Barcs against removal‐directed attack based on betweenness, degree, and cascading. (b) Comparison of the effect of node addition (*n* = 500) by measuring APL and LCC size between Pécs and Barcs groups.

**Figure:**
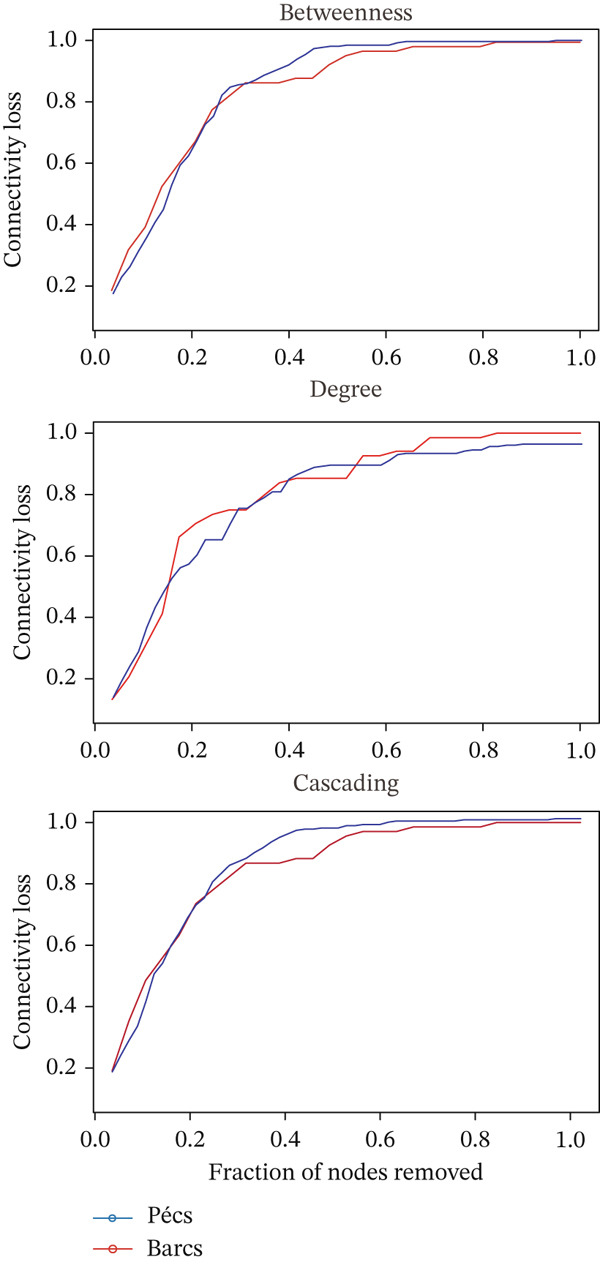
(a)

**Figure:**
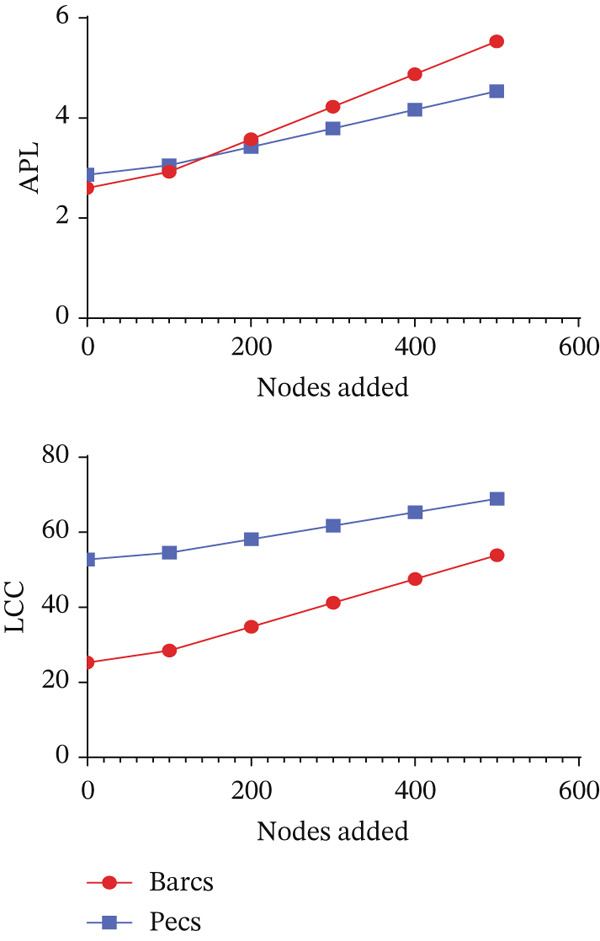
(b)

The APL was higher in Barcs (Figure 4b); this indicates that the microbial network is more dispersed, with taxa being more distantly connected than in Pécs. Pécs network is more efficiently connected when new nodes are added. The LCC (Figure 4b) is higher in Pécs, meaning that when nodes are added, Pécs maintains a larger connected microbiome network than Barcs.

These results suggest important biological implications for the stability and resilience of the analyzed microbiomes. The Barcs network, despite requiring more node deletions to achieve disconnection under certain scenarios, shows signs of structural fragility due to its limited redundancy and longer APLs. This implies that microbial taxa in Barcs are less integrated, making the community more vulnerable to perturbations and less able to maintain functional interactions under stress. In contrast, the Pécs network′s shorter path lengths and larger connected component reflect a more cohesive and adaptable microbiome, potentially more resilient to ecological perturbations to preserve microbial functions.

### 3.4. Differential Influence of the Removal of Shared Taxa on Microbial Assembly and Stability

To assess the structural significance of shared bacterial genera, four key taxa were removed from the microbial networks of both mosquito populations (*Pantoea*, *Stenotrophomonas*, *Delftia*, and *Alcaligenes*). Using topological variation and robustness analysis, the differential impact of this perturbation on the structure, nestedness, and stability of the microbial community associated with mosquitoes from the Pécs and Barcs regions was compared in the presence and after removal of shared taxa.

**Figure 5:**
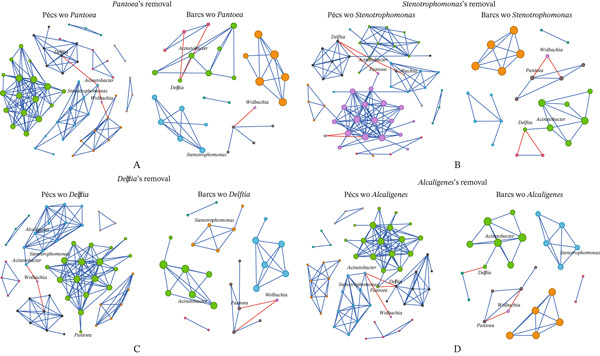
Influence of the removal of shared taxa on microbial assembly. Network analysis of the variation of microbial co‐occurrence patterns in mosquito samples from Pécs and Barcs after the removal of (A) *Pantoea* (woP), (B) *Stenotrophomonas* (woS), (C) *Delftia* (woD), and (D) *Alcaligenes* (woA). Node colors are based on modularity class metric, whereas the size of the nodes is proportional to the eigenvector centrality value of each taxon. The colors of the edges represent strong positive (blue) or negative (red) correlations (SparCC > 0.5 or < −0.5).

Following the targeted removal of the four conserved bacterial genera—*Pantoea* (Figure 5A; Table 3), *Stenotrophomonas* (Figure 5B; Table 3), *Delftia* (Figure 5C; Table 4), and *Alcaligenes* (Figure 5D; Table 4)—the reconstructed Pécs and Barcs networks retained the same broad topological patterns observed in their respective original, unaltered microbial co‐occurrence networks (Figure 2a; Table 1). In both the original (Table 1) and post‐removal (Tables 3 and 4) comparisons, the Barcs network consistently exhibited lower complexity, with fewer microbial interactions, greater compaction, and higher fragmentation (Figures 5A, 5B, 5C, and 5D). These characteristics indicate reduced ecological stability and a limited capacity to recover from external disturbances.

**Table 3:** Topological features of the taxonomic networks of each ecoregion after the removal of shared bacterial genera (*Pantoea* and *Stenotrophomonas*).

Network features	Pécs wo *Pantoea*	Barcs wo *Pantoea*	Pécs wo *Stenotrophomonas*	Barcs wo *Stenotrophomonas*
Number of connected nodes	58	29	58	26
Number of edges	158	43	149	37
Positive interactions	155 (98.1%)	40 (93%)	144 (96.6%)	33 (89.2%)
Negative interactions	3 (1.9%)	3 (7%)	5 (3.4%)	4 (1.2%)
Modularity	0.702	0.798	0.723	0.813
Network diameter	7	5	8	4
Average degree	5.448	2.966	5.138	2.846
Weighted degree	3.246	1.698	2.941	1.551
Clustering coefficient	0.802	0.761	0.781	0.818

**Table 4:** Topological features of the taxonomic networks of each ecoregion after the removal of shared bacterial genera (*Delftia* and *Alcaligenes*).

Network features	Pécs wo *Delftia*	Barcs wo *Delftia*	Pécs wo *Alcaligenes*	Barcs wo *Alcaligenes*
Number of connected nodes	54	28	57	26
Number of edges	158	40	147	40
Positive interactions	156 (98.7%)	38 (95%)	144 (98%)	37 (92.5%)
Negative interactions	2 (1.3 %)	2 (5%)	3 (2%)	3 (7.5%)
Modularity	0.702	0.769	0.723	0.789
Network diameter	7	3	8	5
Average degree	5.852	2.857	5.158	3.077
Weighted degree	3.551	1.724	3.938	1.771
Clustering coefficient	0.849	0.821	0.77	0.782

A key difference from the original networks (Figure 2a; Table 1) was the emergence of negative interactions that had not been present before removal (Figures 5A, 5B, 5C, and 5D; Tables 3 and 4). This shift may reflect intensified competition among the remaining taxa for overlapping ecological niches, coupled with the breakdown of cooperative relationships. The appearance of these previously absent negative associations suggests potential competitive exclusion or inhibitory signaling as strategies for maintaining dominance within the community.

Interestingly, *Wolbachia* and *Acinetobacter* appeared exclusively in the reconstructed networks after the removal of the four conserved genera, despite being absent from the original network structures. In both cases, their interactions were exclusively negative, underscoring the potential for highly abundant taxa to drive community reorganization once key structural members are lost. Their late emergence may represent a compensatory shift in which dominant taxa adopt antagonistic roles to stabilize the network under constrained conditions. Alternatively, their absence in the original networks could reflect selective pressure from other dominant taxa through competitive exclusion or functional redundancy, limiting their need or opportunity to engage in direct interactions within the intact community.

In addition to *Stenotrophomonas* (Figure 5B; Table 3), these results identify *Pantoea* (Figure 5A; Table 3), *Delftia* (Figure 5C; Table 4), and *Alcaligenes* (Figure 5D; Table 4) as potential compensatory taxa capable of assuming more central structural roles in the microbial community when other core members are lost.

**Figure 6:**
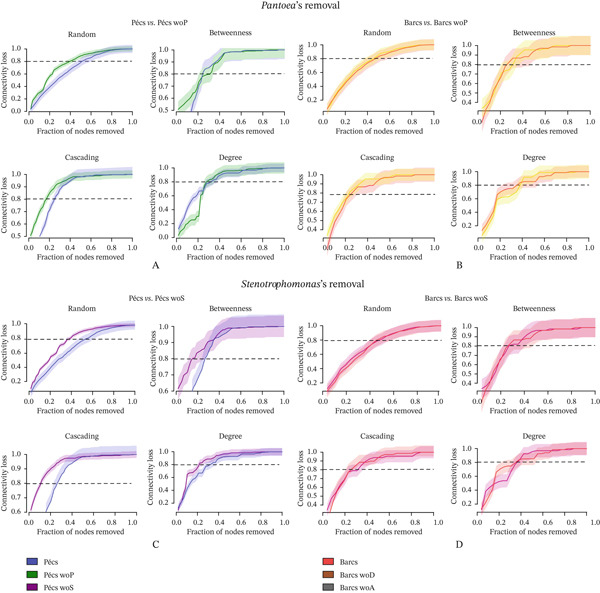
Robustness comparison against removal of nodes between Pecs and Barcs in the presence and after removal of shared taxa. (A) Comparison between Pécs vs. Pécs wo *Pantoea* (woP) against removal directed attack based on random, betweenness, degree, and cascading attack. (B) Comparison between Barcs vs. Barcs wo *Pantoea* (woP) against removal directed attack based on random, betweenness, degree, and cascading attack. (C) Comparison between Pécs vs. Pécs wo *Stenotrophomonas* (woS) against removal directed attack based on random, betweenness, degree, and cascading attack. (D) Comparison between Barcs vs. Barcs wo *Stenotrophomonas* (woS) against removal directed attack based on random, betweenness, degree, and cascading attack.

**Figure 7:**
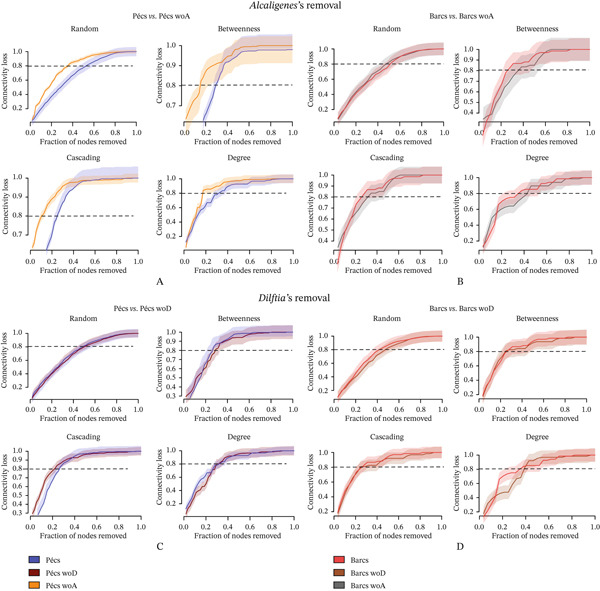
Robustness comparison against removal of nodes between Pecs and Barcs in the presence and after removal of shared taxa. (A) Comparison between Pécs vs. Pécs wo *Alcaligenes* (woA) against removal directed attack based on random, betweenness, degree, and cascading attack. (B) Comparison between Barcs vs. Barcs wo *Alcaligenes* (woA) against removal directed attack based on random, betweenness, degree, and cascading attack. (C) Comparison between Pécs vs. Pécs wo *Dilftia* (woD) against removal directed attack based on random, betweenness, degree, and cascading attack. (D) Comparison between Barcs vs. Barcs wo *Dilftia* (woD) against removal directed attack based on random, betweenness, degree, and cascading attack.

Network robustness—defined here as the capacity of the network to endure disturbances with minimal loss of connectivity—was evaluated under four disturbance scenarios: random node removal, betweenness‐based removal, cascading failure, and degree‐based removal. Robustness was quantified as network connectivity loss in response to increasing fractions of node removal. This analysis compared the reconstructed networks (after removal of *Pantoea*, *Stenotrophomonas*, *Alcaligenes*, or *Delftia*) with the original networks of Pécs and Barcs (Table 1), analyzed independently. The impact of *Pantoea*′s removal (Figure 6a,b) was more pronounced in Pécs than in Barcs across both directed and random attack scenarios, indicating that *Pantoea* plays a more significant structural role in maintaining stability and cohesion in the Pécs microbial community. A similar pattern was observed for *Stenotrophomonas* (Figure 6c,d) and *Alcaligenes* (Figure 7a,b), whose removal also led to greater destabilization in Pécs compared with Barcs. In contrast, the removal of *Delftia* (Figure 7c,d) did not produce marked differences between the two networks, suggesting a more neutral or redundant role for this genus in terms of structural contribution.

Overall, these results demonstrate that while some shared bacterial genera contribute critically to maintaining network integrity and resilience—particularly in Pécs—others may have less direct influence on structural stability, potentially due to functional redundancy within the microbial community.

## 4. Discussion

In this study, we characterized the bacterial microbiome of the invasive mosquito *Ae*. *albopictus* in Hungary, comparing populations from two urban environments (Pécs and Barcs) using Oxford Nanopore sequencing. Our results provide novel insights into the local microbial community structure, diversity, and network stability of this important disease vector, which may influence vector competence and support the development of innovative microbiome‐based vector control strategies. Although our sample size seems quite low, which may limit statistical power, our sequencing depth and data quality ensured sufficient coverage to capture the dominant bacterial taxa, allowing reliable comparative analyses within the exploratory scope of this work. The microbiome composition of *Ae*. *albopictus* showed substantial geographic variation, with marked asymmetry between study locations. Alpha diversity indices showed consistently higher values in Pécs, although they did not reach statistically significant results. This pattern of locality‐specific bacterial communities aligns with observations from various global regions—including Spain, São Tomé, Panama, South Korea, China, and Thailand—where *Ae. albopictus* consistently demonstrates limited core microbiota, predominantly influenced by local environmental conditions, habitat characteristics, and host‐related factors [25, 48–51].

Beta diversity analyses indicated partial but incomplete separation between the two Hungarian populations, consistent with Bennett et al.′s observation that both *Aedes aegypti* and *Ae*. *albopictus* share a large proportion of their microbiota, but with high variability and stochasticity at the local level [48]. Our taxonomic analysis confirmed the overwhelming dominance of *Wolbachia* in both Hungarian populations [25], aligning with global studies. A dual role of *Wolbachia* in manipulating mosquito reproduction and blocking pathogen transmission was emphasized by Shi et al. [52], which has led to substantial reductions in arbovirus incidence in field trials [52]. However, this contrasts with Bennett et al. [48], who found a low incidence of natural *Wolbachia* infection in Panamanian *Aedes* populations, highlighting the regional variability in *Wolbachia* prevalence and its implications for vector competence and biocontrol strategies.

The mosquito microbiome can profoundly influence vector competence for arboviruses, either enhancing or suppressing pathogen transmission [53]. Mechanisms include immune priming, production of antiviral metabolites, resource competition, and regulation of host miRNAs. For example, bacteria such as *Chromobacterium* and *Proteus* have been shown to reduce DENV replication in *Aedes* mosquitoes, whereas antibiotic treatment or environmental perturbation of the microbiome can alter susceptibility to viral infection. On the other hand, arbovirus infection can reciprocally alter the mosquito microbiome, as seen with CHIKV infection in *Ae. albopictus*, which increases *Enterobacteriaceae* abundance and reduces *Wolbachia*. This dynamic interplay underscores the importance of considering both directions of influence—microbiome on pathogen and pathogen on microbiome—when evaluating vector competence and control strategies [53]. Although we did not assess the presence of arboviruses in the analyzed mosquito individuals, based on available public health data, there is no evidence of pathogen circulation or reported mosquito‐borne infections in the studied areas. Hence, our findings of strong local differentiation and distinct network architectures suggest that such functional effects may vary regionally, and that network stability and the presence of key taxa could influence the efficacy of microbiome‐based interventions [53].

Besides the composition, the structure of the mosquito microbiome is also important in the alteration of vector competence, with community stability and interactions potentially affecting both immune priming and resource competition [30]. Such structural features can impact not only pathogen‐blocking efficiency but also mosquito fitness traits like metabolism, blood digestion, and egg‐production traits that are increasingly recognized as microbiome dependent [52]. In the co‐occurrence networks, highly dominant taxa such as *Wolbachia* did not appear because SparCC detects covariation rather than absolute abundance. Dominant genera with low variance across samples do not form statistically significant correlations and are therefore excluded from the network, which is a characteristic of correlation‐based network inference where connectivity is driven by variability rather than abundance. Our network analyses also demonstrate that not only the composition, but structure and stability of microbial communities differ locally. The more cohesive, interconnected network in Pécs may reflect a microbiome with greater functional redundancy and resilience, whereas the fragmented Barcs network could be more susceptible to perturbation. Besides abiotic factors and local environmental conditions, the observed differences between the two *Ae. albopictus* populations may be influenced by the distinct colonization histories of the sites. Based on local monitoring data, the population of *Ae. albopictus* in Pécs was only recently established (first emergence in late 2022, then expanded rapidly in 2023), which could reflect the early‐stage dynamics of microbiome assembly, where newly introduced hosts are colonized by a broad range of environmental microbes, leading to a more heterogeneous and interactive community structure. Although an established population in Barcs, known to have been present for a longer time (since 2020 at the latest), has likely been exposed to local biotic and abiotic selective pressures for an extended period, resulting in long‐term ecological filtering, where over time, only specific, well‐adapted microbial taxa persist within the host, leading to a more specialized but less diverse microbiome. Although direct evidence for the relationship between the population age and the revealed differences between the investigated populations is lacking, such processes are consistent with microbial community maturation and host‐microbe coadaptation, potentially reducing the number of co‐occurring taxa and weakening overall network cohesion. These findings support the hypothesis that population age and colonization history can significantly influence the structure and complexity of mosquito‐associated microbial communities [54, 55], indeed, this hypothesis requires future confirmation.

Robustness analyses show that both networks are similarly sensitive to targeted node removal, but the Pécs network maintains a larger connected component and more efficient connectivity when new taxa are added. This suggests that Pécs′ microbiome may be more robust to ecological disturbances—a feature that could influence the efficacy of microbiome‐based interventions, as highlighted by Ferreira et al. [30] and Shi et al. [52].

On the other hand, comparative analysis of the microbial networks of Pécs and Barcs following the selective removal of four conserved bacterial genera highlights the specific contributions of each taxon to community stability. Microbial community stability can be determined by ecological dependencies between specific taxa, the intensity of which varies depending on network structure and taxon functionality [56–58]. In the more interconnected Pécs network, the removal of *Pantoea*, *Stenotrophomonas*, and *Alcaligenes* led to pronounced destabilization, suggesting a possible role as structural keystone taxa. This suggests a high degree of ecological dependence, in contrast to the lower complexity and fragmentation of the Barcs network, which resulted in lower sensitivity but limited adaptive flexibility. The exclusive emergence of *Wolbachia* and *Acinetobacter* with negative interactions following taxon removal points to patterns of latent dependence, possibly determined by competitive exclusion mechanisms within the original community.

Both Shi et al. [52] and Ferreira et al. [30] review how the mosquito microbiome—especially *Wolbachia*—modulates vector competence through immune activation, direct pathogen interference, and resource competition. Our findings suggest that these functional roles may vary regionally, affecting both vector competence and the success of interventions such as *Wolbachia* releases or antimicrobiota vaccines. The potential for microbiome manipulation is further supported by recent studies showing that targeted interventions can shift mosquito microbiota, impacting both pathogen transmission and mosquito fitness.

## 5. Conclusions

This study presents the first high‐resolution, full‐length 16S rRNA gene profiling of *Ae*. *albopictus* microbiomes in Hungary using Oxford Nanopore sequencing. Despite the relatively short distance between the two sampling sites, Pécs and Barcs, the results revealed substantial differences in the composition, diversity, and organization of their bacterial communities. Although both mosquito populations were dominated by *Wolbachia*, there was limited genus‐level overlap beyond this common symbiont, indicating the presence of largely distinct microbial consortia.

The microbiome of *Ae. albopictus* from Pécs exhibited greater taxonomic richness, more complex network topology, and higher cohesion compared with Barcs, suggesting greater ecological robustness and potential functional redundancy. In contrast, the Barcs population displayed a sparser and more fragmented microbiome structure, which may indicate a more stable but less resilient microbial community. These site‐specific differences, even across a 65 km gradient, underscore the dynamic nature of mosquito‐associated microbiota and highlight the importance of local ecological context.

Such findings have important implications for understanding the microbiome′s role in mosquito biology and vectorial capacity. They also support the need for tailored, location‐specific microbiota‐based vector control strategies in Central Europe. The baseline data provided here pave the way for future functional studies linking microbiome structure to pathogen transmission and mosquito fitness under varying environmental conditions.

## Author Contributions

**Kornélia Kurucz:** conceptualization, methodology, resources, writing – review & editing. **Alejandro Cabezas-Cruz:** conceptualization, supervision, writing – review & editing. **Lianet Abuin-Denis:** methodology, writing – review & editing. **Camille Philippe:** formal analysis, methodology, writing – original draft, visualization, writing – review & editing. **Elianne Piloto-Sardiñas:** formal analysis, writing – original draft, visualization, writing – review & editing. **Myriam Kratou:** formal analysis, writing – review & editing. **Ágota Ábrahám:** investigation, writing – review & editing. **Andrea Kovács-Valasek:** investigation, writing – review & editing. **Dasiel Obregon:** software, writing – review & editing. **Kornélia Kurucz and Camille Philippe** contributed equally to this work.

## Funding

This study was supported by National Research, Development and Innovation Office, Hungary (Nos. FK‐138563 and RRF‐2.3.1‐21‐2022‐00006) and University of Pécs, Hungary.

## Ethics Statement

Ethical review and approval were not required for this study, as it involved the collection and analysis of *Aedes albopictus* mosquitoes, which are not subject to animal experimentation regulations under current institutional and national guidelines.

## Consent

The authors have nothing to report.

## Conflicts of Interest

The authors declare no conflicts of interest.

## Supporting Information

Additional supporting information can be found online in the Supporting Information section.

## Supporting information


**Supporting Information 1** Figure S1: Alpha rarefaction curves of mosquito microbiome samples.

**Supporting Information 2** Table S1: Taxonomic classification and read counts per mosquito sample.

**Supporting Information 3** Table S2: Microbial composition of the mosquito microbiome from Pécs and Barcs.

## Data Availability

The data that support the findings of this study are openly available in Sequence Read Archive (SRA) at https://www.ncbi.nlm.nih.gov/sra, Reference Number PRJNA1290917.
