# Supplementary figures and images for: Characterizing the Bacterial Microbiome of the Invasive Vector Aedes albopictus in Hungary: A Pilot Study Using Oxford Nanopore Sequencing
Source: Int J Microbiol. 2026 Feb 18;2026:1956331. doi: 10.1155/ijm/1956331 (PMC12916851; doi:10.1155/ijm/1956331)

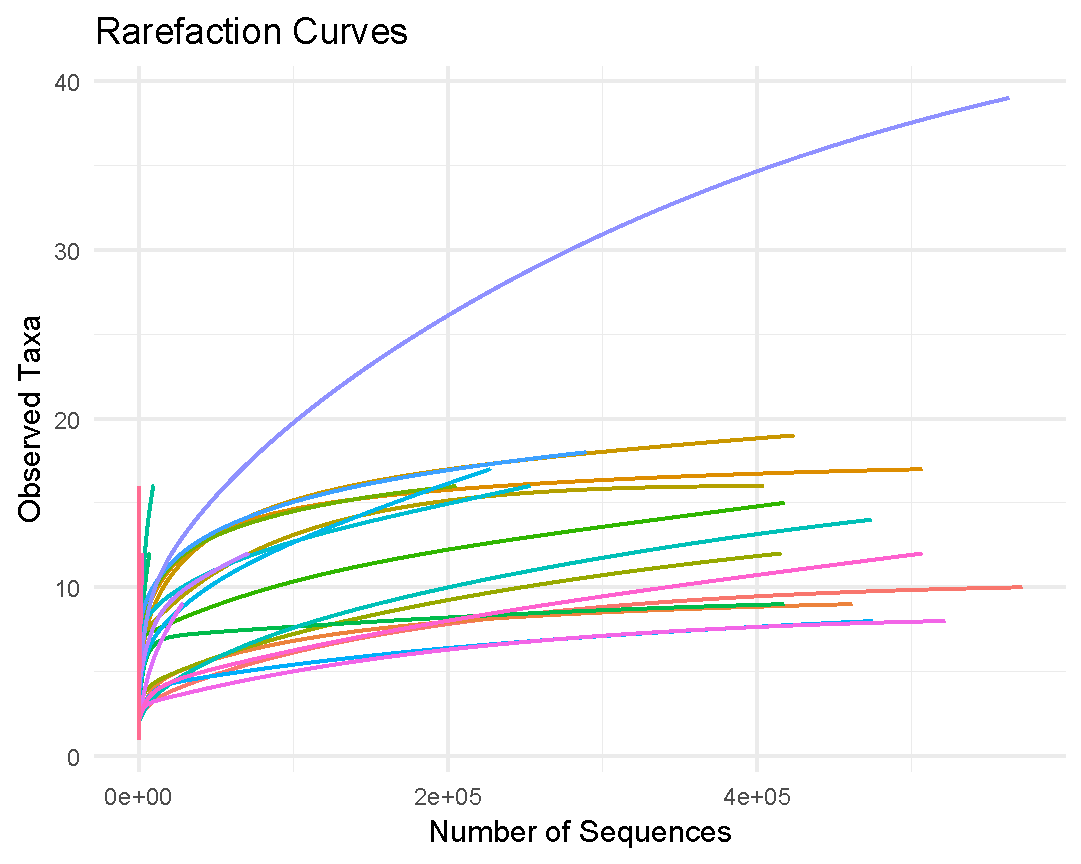

Supplement: Supplementary file 1 — Supporting Information 1 Figure S1: Alpha rarefaction curves of mosquito microbiome samples. [file IJM-2026-1956331-s001.png]
